# Comparison of Immunogenicity Between a Candidate Live Attenuated Vaccine and an Inactivated Vaccine for Cache Valley Virus

**DOI:** 10.1089/vim.2022.0103

**Published:** 2023-01-16

**Authors:** Victoria B. Ayers, Yan-Jang S. Huang, Alain Kohl, James I. Dunlop, Susan M. Hettenbach, So Lee Park, Stephen Higgs, Dana L. Vanlandingham

**Affiliations:** ^1^ Department of Diagnostic Medicine/Pathobiology, College of Veterinary Medicine, Kansas State University, Manhattan, Kansas, USA.; ^2^ Biosecurity Research Institute, Kansas State University, Manhattan, Kansas, USA.; ^3^ MRC-University of Glasgow Centre for Virus Research, Glasgow, Scotland, United Kingdom.

**Keywords:** Cache Valley virus, live attenuated vaccine, inactivated vaccine, sheep

## Abstract

Cache Valley virus (CVV) is a mosquito-borne bunyavirus that is enzootic throughout the new world. Although CVV is known as an important agricultural pathogen, primarily associated with embryonic lethality and abortions in ruminants, it has recently been recognized for its expansion as a zoonotic pathogen. With the increased emergence of bunyaviruses with human and veterinary importance, there have been significant efforts dedicated to the development of bunyavirus vaccines. In this study, the immunogenicity of a candidate live-attenuated vaccine (LAV) for CVV, which contains the deletion of the nonstructural small (NSs) and nonstructural medium (NSm) genes (2delCVV), was evaluated and compared with an autogenous candidate vaccine created through the inactivation of CVV using binary ethylenimine (BEI) with an aluminum hydroxide adjuvant (BEI-CVV) in sheep. Both 2delCVV and BEI-CVV produced a neutralizing antibody response that exceeds the correlate of protection, that is, plaque reduction neutralization test titer >10. However, on day 63 postinitial immunization, 2delCVV was more immunogenic than BEI-CVV. These results warrant further development of 2delCVV as a candidate LAV and demonstrate that the double deletion of the NSs and NSm genes can be applied to the development of vaccines and as a common attenuation strategy for orthobunyaviruses.

## Introduction

Cache Valley virus (CVV) is an emerging zoonotic mosquito-borne orthobunyavirus (family *Peribunyaviridae*, order *Bunyavirales*). CVV was first isolated in Cache Valley, Utah, USA, in 1956 from *Culiseta inornata* (Holden and Hess, 1959). Since then, CVV has become endemic in Canada, Mexico, and the United States, where the virus circulates in mosquitoes and mammals such as white-tailed deer, sheep, goats, and cattle (Armstrong et al, 2015). Although humans are dead-end hosts, if infected, CVV can cause rare but severe disease (Campbell et al, 2006; Nguyen et al, 2013; Wilson et al, 2017; Yang et al, 2018).

Historically, CVV has been recognized as an agricultural pathogen causing embryonic and fetal death, neonatal malformations, and abortions in ruminants, resulting in significant economic loss (Chung et al, 1991; Meyers et al, 2015; Uehlinger et al, 2018). Despite its importance in the livestock industry, there are no licensed vaccines available to prevent or control CVV.

Like other orthobunyaviruses, the CVV genome comprises three negative-sense RNA segments, small (S), medium (M), and large (L), which code for various structural and nonstructural (NS) proteins (Hughes et al, 2020). The S segment encodes the nucleocapsid (N) protein and an alternative overlapping reading frame for the nonstructural protein, nonstructural small (NSs). The M segment encodes two glycoproteins (Gn and Gc) and the other nonstructural protein, nonstructural medium (NSm). The L segment encodes the RNA-dependent RNA polymerase (RdRp). The genome segments are encapsidated by the N protein, which is associated with the RdRp to form ribonucleoprotein complexes (RNP) termed nucleocapsids (Dunlop et al, 2018).

The RNPs are then packaged in the lipid bilayer of the viral membrane, which contains Gn and Gc glycoproteins. The NSs protein has been linked to the virulence phenotype of CVV (Dunlop et al, 2018), whereas the NSm protein is also a putative virulence factor for bunyaviruses (Eifan et al, 2013).

The function of the NSs protein has been evaluated in several orthobunyaviruses, including CVV, Kairi virus, Oropouche virus, Akabane virus (AKAV), Bunyamwera virus (BUNV), Schmallenberg virus (SBV), and La Crosse virus (Blakqori et al, 2007; Bridgen et al, 2001; Dunlop et al, 2018; Ishihara et al, 2016; Tilston-Lunel et al, 2015). Although found to be nonessential for viral growth in cell culture, the NSs protein is a type-I interferon antagonist and has the ability to modulate apoptosis of infected cells, which is part of the host immune responses (Blakqori et al, 2007; Eifan et al, 2013; Kohl et al, 2003).

Although the NSm protein has been studied less extensively than the NSs protein, it has been shown to be associated with viral infection and replication (Leventhal et al, 2021). The function of the NSm protein differs from that of the NSs protein since it does not impair a virus's ability to infect mammalian cells (Shi et al, 2006). However, experiments have demonstrated that the lack of NSm in BUNV leads to immature viral particle accumulation, signifying a potential role in viral assembly (Fontana et al, 2008).

Deletion of NSm in Rift Valley fever virus (RVFV), a phlebovirus, can lead to lower infection and dissemination rates in mosquitoes compared with the wild type (wt) virus (Crabtree et al, 2012). In addition, AKAV and BUNV without a functional NSm protein had impaired growth in both mammalian and insect cells (Ishihara et al, 2016; Shi et al, 2006).

Although the single deletion of NSs or NSm can attenuate the virulence phenotype of RVFV, the deletion of both the NSs and NSm proteins has the most significant attenuating effect (Crabtree et al, 2012). Several groups have developed a reverse genetics system to generate a recombinant virus that lacks both the NSs and NSm proteins (Bird et al, 2011; Brennan et al, 2011; Crabtree et al, 2012). This method potentially creates a safe and immunogenic live-attenuated vaccine (LAV) without the risk of reversion. A RVFV vaccine generated using this technique was previously determined to be safe and effective in pregnant and nonpregnant animals (Bird et al, 2011).

RVFV lacking both NSs and NSm was also unable to infect *Aedes aegypti*, creating the ideal LAV due to the lack of its ability to infect and be transmitted by mosquitoes (Crabtree et al, 2012). The same approach has been used to delete NSs and NSm in SBV, creating promising candidates for the development of safe and effective SBV veterinary vaccines (Kraatz et al, 2015).

Although LAVs are often more efficient in both the onset of immunity and duration of immunity, autogenous inactivated vaccines can be approved by veterinarians when no commercially licensed vaccine is available. Inactivated vaccines are considered safe and useful tools to prevent the spread of emerging diseases. Several inactivated vaccines have been developed against AKAV and SBV with the ability to induce neutralizing antibodies and prevent viremia after a challenge infection (Kim et al, 2011; Wernike et al, 2013).

Previously, Dunlop et al used a reverse genetics system for CVV to produce recombinant viruses, including rCVVdelNSs (Dunlop et al, 2018). Growth curves were determined and compared for mutant CVV lacking NSs and the wt CVV strain (Dunlop et al, 2018). The mutant virus and wt strain grew at comparable rates in BHK-21 cells, Vero E6 cells, A549 NPro cells, and Aag2 cells. However, the mutant virus grew more slowly than the wt strain in A549 cells and SFT-R cells. They also confirmed that the mutant virus was attenuated across several cell lines, and IFN protection assays confirmed the role of the CVV NSs protein as a type 1 interferon antagonist in mammalian cells (Dunlop et al, 2018).

In this study, the deletion of the NSs and NSm genes was used to further attenuate the virulence phenotype and produce a candidate (2delCVV) LAV. The immunogenicity of the 2delCVV candidate vaccine was evaluated in sheep and then compared with the autogenous vaccine generated by the inactivation of CVV with binary ethylenimine (BEI-CVV) based on serum neutralizing titers in sheep.

## Materials and Methods

### Viruses and cell lines

African green monkey kidney epithelial Vero76 cells were maintained in Leibovitz's L-15 media (Thermo Fisher Scientific, Waltham, MA) supplemented with 10% fetal bovine serum (Thermo Fisher Scientific), 10% tryptose phosphate broth (Sigma-Aldrich, St. Louis, MO), penicillin/streptomycin (Thermo Fisher Scientific), and l-glutamine (Thermo Fisher Scientific) as previously described (Huang et al, 2015). The cells were cultured at 37°C and used for propagation of virus stocks and titration of homogenized tissues as previously described (Huang et al, 2015).

To create the 2delCVV candidate vaccine, a reverse genetics system of CVV was used (Dunlop et al, 2018). As previously described, the deletion of the NSs gene was originally achieved by introducing two stop codons in the open reading frame (ORF) encoding the NSs protein (Dunlop et al, 2018). To further attenuate the virulence phenotype of CVV, nucleotides 1,039–1,476 that encode the ORF of the NSm gene were deleted using site-directed mutagenesis. Hence, the resulting recombinant 2delCVV no longer expresses the NSs and NSm genes. The prototype 6V633 wt strain of CVV, originally isolated from infected *Cs. inornata* in Cache Valley, Utah, in 1956 (Holden and Hess, 1959), was obtained from the collection in the laboratory of Dr. Richard M. Elliot (Watret et al, 1985).

The CVV 6V633 wt strain was used for the autogenous BEI inactivated vaccine candidate. The protocol used for inactivating a virus using BEI was as previously described for porcine reproductive and respiratory syndrome virus (Bahnemann, 1990). In brief, a stock of CVV 6V633 strain at 10^7^ plaque forming unit (pfu)/mL was inactivated, and the complete inactivation was confirmed by the lack of infectivity detected by plaque assay (Baer and Kehn-Hall, 2014; Nuckols et al, 2015).

### Animal experiment and design

The following experimental procedures and handling of live animals were approved by the Kansas State University Institutional Animal Care and Use Committee. All methods were carried out in accordance with the approved protocol and relevant regulations. All animal work was conducted in the large animal research center in biosafety level 2 agriculture (BSL2-Ag) conditions. Animals were allowed an acclimation period of 5 days in the BSL2-Ag housing before the start of the experiments. Throughout the experiment, all animals were given *ad libitum* access to fresh water and fed a commercial grade pellet ration according to the animal's body weight.

The objective of this experiment was to evaluate and compare the immunogenicity of a candidate LAV for CVV and an autogenous inactivated vaccine for CVV. After the primary immunization, boosters on days 21 and 42 were used to determine whether either vaccine increased the titers of neutralizing antibodies that would likely provide a protective immune response. Twenty-four 6-month-old male Rambouillet lambs were first assigned to one of the following vaccine groups: the 2delCVV vaccine group (*n* = 10), the BEI-CVV group (*n* = 10), or the L-15 media group (*n* = 4).

Before initial immunization, all animals were determined to be healthy and seronegative to CVV through the analysis of collected serum using 50% plaque reduction neutralization tests (PRNT_50_). On day 0 of the experiment, lambs were immunized subcutaneously at their fore flank (right behind their elbow) with their corresponding immunization regimen, including (1) 1 mL 2delCVV containing 10^5^ pfu of infectious viruses, (2) 1 mL BEI-CVV with the addition of the aluminum hydroxide adjuvant, or (3) 1 mL of L-15 media.

The inoculum of BEI-CVV contained equal concentration of virus particles corresponding to viral stocks at 5 × 10^6^ pfu/mL based on the equal volume mixture of inactivated virus stock and BEI. Lambs then received booster immunizations on days 21 and 42 postinitial immunization. Four milliliters of blood was collected from each animal on days 3, 5, 7, 14, 20, 35, 41, 56, and 63 postinitial immunization. On the days blood was collected, the blood volume did not exceed 1% of the total blood for each animal. Serum samples were then obtained through the centrifugation of the coagulated blood at 2,000 × *g* for 10 min at 4°C and stored in a −80°C freezer for later analysis.

**FIG. 1.**
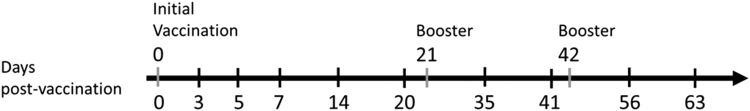
Timeline of experimental design. Rambouillet ram lambs (L-15 media, *n* = 4; BEI-CVV, *n* = 10; 2delCVV, *n* = 10) were immunized subcutaneously on day 0 with booster immunizations administered on days 21 and 42 postinitial immunization. Serum samples were collected on days 0, 3, 5, 7, 14, 20, 35, 41, 56, and 63 of the study for the assessment of neutralizing antibody activity. BEI, binary ethylenimine; CVV, Cache Valley virus.

The experiment ended 63 days postinitial immunization and the sheep were euthanized. For the duration of the study, animals were monitored daily for any clinical signs including fever (>40°C), depression, weight loss, respiratory distress, lameness, neurological signs, and vaccine site reactions. Figure 1 illustrates the timeline of the experiment.

### Plaque reduction neutralization test

To determine the neutralizing antibody titers, PRNT_50_ was performed as previously described (Roehrig et al, 2008). All serum samples were first heat inactivated at 56°C for 30 min and twofold serial dilutions between 1:5 and 1:160 were tested (OIE, 2014). Approximately 30 pfu of the CVV 6V633 strain was added to each serum concentration and incubated for 1 h at 37°C before infection of Vero76 cells in 24-well plates. The wells were then gently washed with Dulbecco's phosphate-buffered saline and overlaid with 1% methylcellulose. After 5 days of incubation at 37°C, the wells were fixed with 10% formalin solution and then stained with 1% crystal violet stain. Plaques were then counted, and the neutralizing antibody titers were calculated based on PRNT_50_.

### Statistical analyses

All statistical analyses were conducted using the GraphPad Prism (version 8.1.2) program (GraphPad Software, Inc., San Diego, CA). The Shapiro–Wilk test was used to test for normal distribution. PRNT_50_ titers were compared between the two vaccine groups on each day using Mann–Whitney *U* tests. A two-way analysis of variance (ANOVA) was used to determine the significant difference among and between the candidate vaccine's PRNT_50_ titers from the initial and booster vaccinations. To evaluate the significant difference between the two vaccine groups over the duration of the study, a two-way ANOVA was performed.

## Results

The animals were healthy and seronegative to CVV before immunization. Animals from both the live attenuated candidate 2delCVV vaccine group and the inactivated BEI-CVV vaccine group presented no observable adverse clinical signs, weight loss, or increased rectal temperatures, however, all animals that received BEI-CVV had swelling averaging 3 cm in diameter present at the injection site after vaccination that persisted for the duration of the study. Animals were bled on days 3, 5, 7, and 14 to determine immune responses after the primary immunization, and on days 20, 35, 41, 56, and 63 to determine whether booster immunizations would increase the immunogenicity and produce a long-lasting neutralizing antibody response with each candidate vaccine.

**FIG. 2.**
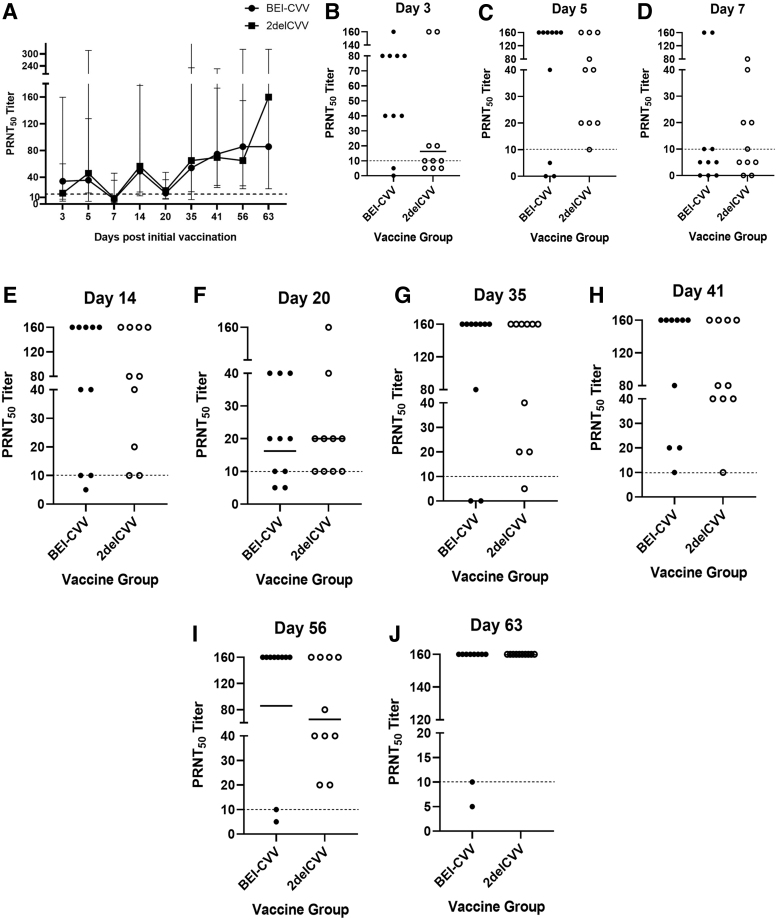
Serum neutralizing activity of sheep receiving 2delCVV and BEI-CVV between 3 and 63 days postinitial immunization. **(A)** Kinetics of serum PRNT_50_ titers between sheep immunized with 2delCVV and BEI-CVV over time were compared using the Mann–Whitney rank sum test. Each subfigure represents the serum PRNT_50_ titers at a specific time point—at day 3 **(B)**, day 5 **(C)**, day 7 **(D)**, day 14 **(E)**, day 20 **(F)**, day 35 **(G)**, day 41 **(H)**, day 56 **(I)**, and day 63 **(J)**. The *dashed line* represents 1:10 serum PRNT_50_ titer, which is the correlate of protection used in arbovirus vaccine clinical trials. Titers of individual animals immunized with 2delCVV and BEI-CVV are shown with *open* and *closed circles*, respectively. *Solid bar* in each subfigure represents the geometric mean of the serum PRNT_50_ titers of sheep receiving 2delCVV or BEI-CVV. PRNT_50_, 50% plaque reduction neutralization tests.

All animals immunized with the candidate LAV seroconverted above the 1:10 PRNT_50_ titer, that is, immune correlate of protection, after a single immunization on day 20 postinitial immunization. There was no significant difference in the neutralizing antibodies elicited by both vaccines when compared (Fig. 2). However, on day 63 postinitial immunization, the 2delCVV candidate vaccine induced a slightly higher neutralizing antibody response than the autogenous vaccine, although it did not reach statistical significance (Fig. 2).

Importantly, immunization of BEI-CVV only led to transient neutralizing antibody responses. The serum PRNT_50_ titers of 20% of animals (2/10) receiving BEI-CVV waned to 5 and 10, whereas all animals receiving 2delCVV maintained robust neutralizing activity with all animals ending the study with PRNT_50_ titers of 160.

## Discussion

In this study, we evaluated the immunogenicity of the candidate 2delCVV LAV and an autogenous CVV vaccine, BEI-CVV. Although both vaccines provided a neutralizing antibody response past the threshold of immune protection, animals immunized with 2delCVV LAV developed higher serum neutralizing titers at 63 days postimmunization. We conclude that 2delCVV is superior in immunogenicity and can be further evaluated as a candidate veterinary LAV.

Inactivated vaccines are generally safe and can be developed within a relatively short period of time, making them a putative counter measure against abrupt outbreaks of viral diseases. The inactivated candidate vaccine used in this study was created similarly to one of the inactivated vaccine candidates used against SBV (Wernike et al, 2013). They were both inactivated with BEI with the addition of an aluminum hydroxide adjuvant. The outcome was also similar with most of the animals developing detectable neutralizing antibodies upon vaccination.

However, as expected, the immunogenicity of the inactivated vaccine is low and leads to the requirement of at least a two-dose immunization regimen to produce sufficiently high serum neutralizing antibodies, the correlate of protection. This indicates that the first dose may only give partial protection and the maximum immune response is not achieved until after one or two booster immunizations. This justifies the development of 2delCVV as a candidate LAV. This study proved the superior immunogenicity of 2delCVV compared with CVV-BEI, suggesting that a similar approach to rationally design candidate LAVs may offer more effective tools for the control of CVV and other related orthobunyaviruses.

A challenge study would be necessary to determine actual protection in pregnant sheep against the fetal malformations and abortions that infection with CVV causes. Such challenges could not be performed during the current studies. Since CVV does not normally display clinical symptoms in adult ruminants, challenging the sheep used in this study would not have given relevant information on protection. Future studies could include a challenge study conducted to examine safety and protection in pregnant ruminants, evaluating the cytokine profile, and determining the vaccine-induced Immunoglobulin G/Immunoglobulin M host antibody response by antigen-specific indirect enzyme-linked immunosorbent assays.

Previously an inactivated vaccine for RVFV reduced viremia with a lack of clinical signs in a vaccinated lamb, however, there were no detectable neutralizing antibodies (Kortekaas et al, 2012). This could potentially mean that the inactivated vaccine could provide adequate protection with only one single immunization without the need for booster immunizations. In addition, the use of adjuvants is sometimes problematic. Aluminum hydroxide adjuvants have been shown to cause granulomas and potentially act as a contributor to severe wasting syndrome (de Miguel et al, 2021; Echeverría et al, 2020).

LAVs are typically considered to be easier to produce and more efficacious. Modified live vaccines were initially based on random introduced mutations through serial passages in cell culture or in the presence of chemical mutagens (Henderson, 2005; Vannie et al, 2007), however, there have been incidences wherein the vaccine will revert to a wt or pathogenic form (Shams, 2005; Weyer et al, 2016). Reassortment between orthobunyaviruses has also been shown to be a major component in orthobunyavirus evolution (Briese et al, 2007). Another limitation is the inability for these vaccines to be used as differentiating infected from vaccinated animals vaccines.

The live attenuated candidate 2delCVV vaccine was created with the inability of the virus to express the NSs and NSm genes. This attenuated the virus so it would elicit an immune response without the ability to revert to full virulence. The complete deletion of genes could possibly enable us to distinguish vaccinated from field-infected animals. This could potentially be achieved through the detection of the wt virus using an NSs or NSm antibody and through detection of the mutant virus using an anti-NSs or anti-NSm antibody as previously described (Bird et al, 2011). A CVV double deletion mutant virus would also have significant advantages similar to the double deletion mutant for SBV (Kraatz et al, 2015), including its potential inability to be transmitted by insect vectors.

Although experiments with BUNV have demonstrated how NSs was nonessential in mosquito cell lines, NSs was shown to be important for the infection of mosquitoes (Szemiel et al, 2012). In addition, NSm is important for virulence in insect hosts (Crabtree et al, 2012). Demonstrating the need for both NSs and NSm for efficient growth of the virus in arthropod cells and suggesting that spread into vector populations is unlikely for 2delCVV.

As expected, the autogenous BEI-CVV vaccine was capable of eliciting a neutralizing antibody response, however, a booster immunization would be needed and there was an adverse reaction at the injection site. The 2delCVV candidate vaccine not only produced a neutralizing antibody response through the duration of the study that could confer protection, but it also produced a more robust neutralizing antibody response at the end of the study when compared with the autogenous vaccine. Therefore, through the deletion of the NSs and NSm genes, an immunogenic vaccine for CVV was developed. Although protection studies are warranted, these data provide a basis for further development of immunogenic vaccines for CVV and other related orthobunyaviruses.
